# Utilizing Participatory Research to Engage Underserved Populations to Improve Health-Related Outcomes in Delaware

**DOI:** 10.3390/nu13072353

**Published:** 2021-07-09

**Authors:** Shannon M. Robson, Samantha M. Rex, Katie Greenawalt, P. Michael Peterson, Elizabeth Orsega-Smith

**Affiliations:** 1 Department of Behavioral Health and Nutrition, University of Delaware, 26 N College Avenue, Newark, DE 19713, USA; srex2@jhmi.edu (S.M.R.); keg5293@psu.edu (K.G.); pmpeter@udel.edu (P.M.P.); eosmith@udel.edu (E.O.-S.); 2 Department of International Health, Bloomberg School of Public Health, Johns Hopkins University, 615 N Wolfe Street, Baltimore, MD 21205, USA; 3 PennState Extension, College of Agricultural Sciences, The Pennsylvania State University, 323 Agricultural Administration Building, University Park, PA 16802, USA

**Keywords:** community-based, participatory research, anthropometrics, fruit and vegetable intake, physical activity, Cooperative Extension

## Abstract

Cooperative Extension is a community outreach program. Despite its large reach, there is a need for the evaluation of changes in health-related outcomes for individuals engaged with Cooperative Extension. A team-based challenge was developed using community-engaged participatory research integrated with Cooperative Extension to encourage healthy eating and physical activity behaviors through Cooperative Extension programming. Thus, the primary purpose of this secondary analysis was to (1) evaluate changes in anthropometric outcomes and (2) evaluate changes in health behavior outcomes. Associations of anthropometric changes and health behavior changes with engagement in the three-month team-based challenge were explored. Anthropometrics were measured using standard procedures, and intake of fruits and vegetables and physical activity were self-reported. Of the 145 participants in the community-engaged participatory research portion of the study, 52.4% (*n* = 76) had complete anthropometrics before and after the team-based challenge and were included in this study. At 3 months, there was a significant reduction in body mass index (−0.3 kg/m^2^, *p* = 0.024) and no significant change in waist circumference (*p* = 0.781). Fruit and vegetable intake significantly increased (+0.44 servings/day, *p* = 0.018). Physical activity did not significantly change based on (1) the number of days 30 or more minutes of physical activity was conducted (*p* = 0.765) and (2) Godin Leisure-Time Exercise Questionnaire scores (*p* = 0.612). Changes in anthropometrics and health behaviors were not associated with engagement in the team-based challenge. Using community-engaged participatory research with community outreach programs, such as Cooperative Extension, can improve health-related outcomes in underserved populations. However, despite a participatory approach, changes in anthropometrics and health behaviors were not associated with engagement in the developed team-based challenge.

## 1. Introduction

Poor dietary habits and low levels of physical activity are often associated with poor health outcomes, including overweight and obesity [1,2,3]. In the USA, approximately 70% of the adult population is classified as being overweight or obese [4]. Racial/ethnic and socioeconomic disparities exist in obesity with Hispanic and Black populations often experiencing a higher prevalence than White and Asian populations, and lower income populations having higher rates of overweight and obesity than higher income populations [5]. While several social, biological and environmental determinants influence overweight and obesity, nutrition and physical activity are considered to be key factors [6]. In particular, a diet of higher quality has been associated with lower risk of overweight and obesity in adults [7,8,9] and consumption of fruits and vegetables contributes to better diet quality [10]. In addition, regular physical activity is important for maintaining and achieving a healthy weight [11].

In 1914, Cooperative Extension was established with the purpose of providing educational outreach services through land-grant institutions dedicated to delivering science-based programs to people, businesses and communities, with a particular focus on programming promoting health [12]. Cooperative Extension has a wide reach across the USA with extension agents in approximately 3000 counties. Cooperative Extension is a trusted source of information within communities and can often provide known and respected connections to local networks and community leaders. Despite its reach, there has been a continuous call for greater engagement across the Extension system [13,14]. Within Cooperative Extension, participatory research has been suggested as one method of increasing community engagement that may lead to more impactful outcomes [15]. Community-engaged participatory research is a technique that can be used to plan community acceptable programs (interventions) as the community engages as an equal partner in the research.

Improvements in health behaviors (including diet and physical activity) associated with participatory research have been well documented [16,17,18,19,20,21]; however, the integration of community-engaged participatory research with Cooperative Extension has been limited. Thus, among a sample of adults engaged with Cooperative Extension in Delaware (USA), the purpose of this secondary analysis was to (1) examine changes in anthropometric outcomes (body mass index and waist circumference) and (2) examine changes in the health behaviors (fruit and vegetable intake and physical activity) of participants with complete anthropometric measures before and after the team-based challenge. Associations between changes in anthropometrics and changes in health behaviors and engagement in the team-based challenge developed through a community-engaged participatory research process were explored.

## 2. Methods

### 2.1. Participants’ Eligibility and Recruitment

Participants were recruited through personal contact among the Cooperative Extension agents, local churches and community groups. Adults (≥18 years-old) living in Kent or New Castle County, Delaware (USA), were eligible to participate in a community-based participatory research study to develop a program for increased engagement in healthy lifestyle behaviors. Of the 145 adults that were involved in the participatory research portion of the study that created the team-based challenge, 76 had complete anthropometric measures at baseline and end of the team-based challenge (three months) and were included in this secondary analysis with the primary purpose of examining changes in anthropometrics. The study was approved by the Institutional Review Board at the University of Delaware. All participants signed informed consent forms.

### 2.2. Study Procedures

Using community-engaged participatory research, input from community members was collected through key informant interviews, focus groups and community advisory boards. The information collected was used to inform the development of a 3-month team-based challenge. The team-based challenge was created with the purpose of engaging the community in healthy lifestyle behaviors. In teams, anyone from the community could enroll and accrue points each month. Points were awarded based upon participation in a variety of individual and team-based activities. Points were assigned to various health behaviors and activities, such as steps walked per day, parking the car further away, preparing a new recipe, or getting a health screening. Points were allocated based on complexity or effort required to complete an activity; for example, parking the car further away would equate to five points, while walking 10,000 steps a day would equate to 20 points. Participants could also earn points by attending the Cooperative Extension educational programming. Points allocated for participation in these Cooperative Extension programs were based on the number of sessions. For example, participation in Cooperative Extension education sessions, such as “Dining with Diabetes”, would equate to 100 points for multiple class sessions (or partial points if individual classes were attended). Single education session programs, such as “Get Your Snack on Track”, would be worth 50 points. Participants tracked their total points on a log each day, and at the end of the month, team members turned in their logs to the Cooperative Extension health educator, who totaled and averaged the points by number of team members. Participants received incentives, such as gift cards and prizes, as goals were achieved to promote engagement. 

### 2.3. Measures

Anthropometrics were measured and a comprehensive questionnaire, which was available in English and Spanish, that included questions regarding demographics and health conditions, servings of fruit and vegetables and physical activity, was completed by participants at baseline and after the three-month time period of the team-based challenge. 

Anthropometrics. The weight and height of each participant were measured by research personnel using standard procedures [22]. Weight and height were used to calculate body mass index (BMI) by dividing weight in kilograms by the square of height in meters. BMI was used to classify weight status (normal weight: <25 kg/m^2^; overweight: 25–29.9 kg/m^2^; obesity: ≥30 kg/m^2^). The mean of three consecutive waist measurements was taken at the smallest point of the waist [23] in inches and recorded when the tape measure was snug around the waist, but not constricting. Mean waist measurements were converted to centimeter for reporting.

Demographics and Health Conditions. Basic demographic information related to age, education level, marital status and income was collected. Participants self-report (yes or no) of having diabetes, hypertension and/or high cholesterol was collected at baseline.

Servings of Fruits and Vegetables. Fruit and vegetable intake, defined as fresh, frozen, canned, dried, and 100% juice, were assessed based on servings (with one serving equal to a half cup equivalent) with the question “How many fruits and vegetables do you usually eat each day?” Response options ranged from none to five or more servings per day.

Physical Activity. Physical activity was assessed based on the response of 0–7 days to the question “How many days was 30 or more minutes of purposeful physical activity accumulated in a typical week?” and the Godin Leisure-Time Exercise Questionnaire [24]. The Godin Leisure-Time Exercise Questionnaire asks participants “During a typical 7-day period, how many times on average do you do the following kinds of exercise for more than 15 min during your free time?” with response options of ”strenuous exercise” (e.g., running, jogging, hockey, football and soccer), ”moderate exercise” (e.g., fast walking, basketball, tennis, easy bicycling and volleyball), or ”mild exercise” (e.g., yoga, archery, fishing, bowling and golfing). The number of times per option was multiplied by 9, 5 and 3, respectively, then summed to generate a Godin Leisure-Time Exercise score. Scores were categorized into three levels of physical activity: active (≥24 or more), moderately active (14–23), or insufficiently active/sedentary (<14) [25].

Engagement. Engagement was measured through the total number of points accumulated by teams each month in this team-based challenge. Participants earning one or more points over the course of the three-month team-based challenge were considered engaged, but if an individual did not participate or earned zero points, they were considered to have not engaged.

### 2.4. Statistical Analysis

Analyses were conducted using SPSS (IBM, Chicago, IL, USA, version 26). Means and frequencies were used to analyze continuous and categorical data, respectively, for demographics, engagement, anthropometrics, fruit and vegetable intake and physical activity. Paired t-tests examined changes from baseline to three months for anthropometrics, fruit and vegetable intake and physical activity, as measured by the number of days participants accumulated at least 30 min of physical activity and the Godin Leisure-Time Exercise score. The marginal homogeneity test was used to examine the change in the proportion of individuals in each Godin Leisure-Time physical activity category (active, moderately active and insufficiently active/sedentary) from baseline to three months. Chi-square tests were conducted to determine the association between change in anthropometrics, change in servings of fruit and vegetable and change in the level of physical activity outcomes based on the Godin Leisure-Time Exercise score and engagement (yes vs. no). An *alpha* < 0.05 was considered significant *a priori*.

## 3. Results

Of the 145 individuals who participated in the participatory research portion of the study, 52.4% (*n* = 76) participated in the team-based challenge and had complete anthropometrics measures at baseline and three months. Participants were on average 51.3 ± 17.4 years old, 77.0% female, with 59.2% identifying as non-Hispanic, African American, and 34.2% identifying as Hispanic, White. Of those who reported household income, 45.4% reported an annual household income < USD 20,000 per year. The median reported income in the USA in 2017 (when the study was conducted) was USD 61,372 [26]. Among participants, 23.6% reported having diabetes, 39.2% reported having hypertension and 41.3% reported having high cholesterol (Table 1).

As shown in Table 2, on average, participant weight significantly decreased over the three-month time period (t (75) = −2.405; *p* = 0.019). BMI also significantly decreased over the three-month program (t (75) = −2.298; *p* = 0.024). Waist circumference measurements of participants did not significantly change with participation in a three-month program. Participant (*n* = 74) consumption of daily servings of fruits and vegetables significantly increased over time (t (73) = 2.414, *p* = 0.018). There was no statistically significant change reported by participants for the number of days participants accumulated at least 30 min of physical activity or the Godin Leisure-Time Exercise score over the three months. The proportion of individuals within each level of physical activity based on the Godin Leisure-Time Exercise score did not significantly change over time. Changes in weight, BMI, waist circumference, daily servings of fruit and vegetable intake, and Godin Leisure-Time Exercise score for physical activity were not significantly different between individuals who actively engaged versus those who did not.

## 4. Discussion

Impacts of community-based participatory research on health-related outcomes are less established in the literature [27]. This secondary analysis is one of few reporting on anthropometrics and health behaviors [17,19]. The utilization of community-based participatory research to develop a team-based challenge integrated with Cooperative Extension programming found an improvement in anthropometric outcomes based upon BMI and an increase in fruit and vegetable intake (a health behavior). Despite the decrease in BMI being statistically significant, it may have limited the clinical impact on health outcomes. Recommendations suggest 5–10% weight loss for an improvement in health outcomes; however, these recommendations are heavily based upon intensive lifestyle interventions delivered in clinical or research settings [28]. It is known that the dissemination of lifestyle interventions through community-based programs produce smaller weight losses [29] since the goals of community-level interventions are more often focused on increasing reach or increasing access to interventions [30]. Similarly, while there was a significant increase in fruit and vegetable intake over the three-month program, the change was small, equivalent to about a half serving or a one-quarter cup equivalent. Despite the increase, these data demonstrate that the participants still consumed less than the amounts recommended by the US Dietary Guidelines for Americans [31]. Waist circumference did not significantly change, and longer-term evaluation data are needed to better understand a possible maintenance effect. The physical activity level did not significantly change. The majority of participants were engaging in some type of physical activity upon starting the team-based challenge. While non-significant, there was an increase in the number of participants classified as “active” according to the Godin Leisure-Time Exercise categories. While findings suggest that the team-based challenge, developed through community-based participatory research and integrated with Cooperative Extension programming, can have a positive impact on health-related outcomes, changes in health-related outcomes were not associated with engagement. 

It was unexpected that engagement in the team-based challenge was not correlated with outcomes; however, few studies report on engagement in the literature [32], particularly in community-based programs, limiting the ability to understand this finding in a broader context. Furthermore, the concept or definition of community engagement is inconsistent [33] and has more often focused on engaging the community during the development of interventions and programs, such as the community-engaged research paradigm used to develop the team-based challenge as opposed to engagement in the intervention or program developed [34]. Despite variable definitions, interventions that use community engagement, particularly for vulnerable populations, have been shown to be effective in improving health behaviors [35]. Relying on input to develop programs and interventions from individuals already engaged with established community-based outreach programs, such as Cooperative Extension, may further promote engagement in nutrition and physical activity interventions due to the community’s familiarity with the established program. Zoellner and colleagues [20] found that the process of developing and implementing a walking program through community engaged participatory research and partnership with the Mississippi State Cooperative Extension was successful. 

Due to the high prevalence of overweight and obesity in the USA, many community-based programs, such as Cooperative Extension, aim to encourage healthy behaviors related to eating and physical activity to promote a healthier weight. Fruits and vegetables are a large focus due to under-consumption, particularly among lower socioeconomic groups [6,36] who are served by Cooperative Extension outreach programs. There was a significant increase in fruit and vegetable intake over the three-month program, but the change was small, equivalent to about a half serving or a one-quarter cup equivalent. This small change may diminish any potential clinically meaningful impact on health. Although the increase was similar to many other findings investigating fruit and vegetable intake, these data demonstrate that participants consumed less than that recommended by the Dietary Guidelines for Americans [37].

This study is the result of a team-based challenge developed through community-based participatory research and implemented in the community utilizing an existing community resource, Cooperative Extension. Reporting health outcomes as a result of community-based participatory research is a strength of this study that is often cited as a limitation of this type of work. Study personnel measured height, weight and waist circumference of participants, providing a more accurate measurement than self-reporting. Additional study limitations include the cross-sectional nature of the data not allowing for comparison to a control condition and the use of self-reporting for fruit and vegetable intake and physical activity. These measures of fruit and vegetable intake and physical activity may not accurately reflect actual consumption or engagement in activity. Missing data are an additional limitation of this secondary analysis as of the 76 participants with anthropometric data at both time points, two were missing fruit and vegetable data, and 18 were missing the Godin Leisure-Time Exercise Score. The generalizability of the findings is limited given the results are representative of lower-income adults in the state of Delaware (USA) engaging with Cooperative Extension.

## 5. Conclusions

The integration of programming developed through community-engaged participatory research with established community outreach programs, such as Cooperative Extension, may be a novel way to create programs to improve health outcomes in underserved populations. While changes were small, improvements in weight, BMI and fruit and vegetable intake did occur, indicating the potential beneficial impact of programs developed by the community for the community on healthy lifestyle behaviors. However, despite the use of a community-engaged participatory research process, changes to health-related outcomes were not associated with engagement in the team-based challenge.

## Figures and Tables

**Table 1:** Baseline demographics and health condition characteristics of adults participating in the Cooperative Extension team-based challenge with complete anthropometrics.

Characteristics	*N* = 76 ^1^
	M ± SD or *n* (%)
**Age**, years	51.3 ± 17.4
**Sex** [*n* = 74]	
Male	17 (23.0%)
Female	57 (77.0%)
**Race/Ethnicity**	
Non-Hispanic, Black or African American	45 (59.2%)
Hispanic, White	26 (34.2%)
Other	5 (6.6%)
**Education** [*n* = 73]	
No high school degree	13 (17.8%)
High School or GED degree	22 (30.1%)
Some college	15 (20.5%)
College degree	17 (23.3%)
Postgraduate/professional degree	6 (8.2%)
**Marital Status**	
Married	37 (48.7%)
Widowed	4 (5.3%)
Divorced	10 (13.2%)
Separated	4 (5.3%)
Never married	21 (27.6%)
**Household Income** [*n* = 66]	
<USD 20,000	30 (45.5%)
USD 20,000 to <USD 50,000	27 (40.9%)
>USD 50,000	9 (13.6%)
**Diabetes** [*n* = 72]	
Yes	17 (23.6%)
No	55 (76.4%)
**Hypertension** [*n* = 74]	
Yes	29 (39.2%)
No	45 (60.8%)
**High Cholesterol** [*n* = 75]	
Yes	31 (41.3%)
No	44 (58.7%)

^1^ Sample sizes vary due to missing data for the following variables: sex (*n* = 74), education (*n* = 73), household income (*n* = 66), diabetes (*n* = 72), hypertension (*n* = 74), and high cholesterol (*n* = 75).

**Table 2:** Participant (*n* = 76) ^1^ anthropometrics, fruit and vegetable intake and physical activity measures at baseline and after the 3-month team-based challenge.

	Baseline	3-Months	*p*-Value
	M ± SD or *n* (%)	M ± SD or *n* (%)	
**Height**^2^, m	1.6 ± 0.1	N/A	-
**Weight**, kg	82.9 ± 19.1	82.0 ± 18.3	0.019 *
**Waist Circumference**, cm	98.6 ± 16.1	98.5 ± 15.8	0.781
**Body Mass Index**, kg/m^2^	30.7 ± 7.4	30.4 ± 7.1	0.024 *
**Fruit and Vegetable Intake**, servings/day	2.7 ± 1.4	3.2 ± 1.4	0.018 *
**Physical Activity**, days/week of 30+ minute	3.0 ± 2.1	3.0 ± 2.1	0.765
**Godin Leisure-Time Exercise Score**	28.8 ± 30.8	30.9 ± 28.4	0.612
**Godin Leisure-Time Exercise Categories**			0.376
Insufficiently active/Inactive	20 (34.5%)	18 (31.0%)	
Moderately active	11 (19.0%)	9 (15.5%)	
Active	27 (46.6%)	31 (53.4%)	

^1^ Sample sizes vary due to missing data for the following variables: fruit and vegetable intake (*n* = 74), Godin Leisure-Time Exercise Score and exercise categories (*n* = 58). ^2^ Given an adult population, height was only measured at the pre-assessment. * *p* < 0.05.

## Data Availability

The data presented in this study are not publicly available, but available on request from the corresponding author.
